# Concanavalin A Disrupts the Release of Fibrous Material Necessary for Zygote Formation of a Unicellular Charophycean Alga, *Closterium peracerosum*-*strigosum*-*littorale* Complex

**DOI:** 10.3389/fpls.2016.01040

**Published:** 2016-07-13

**Authors:** Jun Abe, Sachie Hori, Mamiko Sato, Hiroyuki Sekimoto

**Affiliations:** ^1^Department of Chemical and Biological Sciences, Faculty of Science, Japan Women’s University, TokyoJapan; ^2^Division of Material and Biological Sciences, Graduate School of Science, Japan Women’s University, TokyoJapan; ^3^Laboratory of Electron Microscopy, Japan Women’s University, TokyoJapan

**Keywords:** charophycean, *Closterium*, conjugation, gamete, Concanavalin A (Con A), sexual reproduction

## Abstract

The *Closterium peracerosum–strigosum–littorale* (*C. psl.*) complex is the best characterized charophycean alga with respect to the processes of sexual reproduction. We examined the effect of concanavalin A (Con A) on physiological and ultrastructural changes during the conjugation of the *C. psl.* complex. Two heterothallic gametangial cells formed a sexual pair as usual; however, the release of gametes was completely blocked by the addition of Con A. Fluorescein isothiocyanate-labeled Con A bound to the outermost layer of the conjugation papillae of paired cells. In the absence of Con A, the disruption of outer cell walls on the conjugation papillae and the secretion of fibrous materials from the conjugation papillae were observed using a transmission electron microscope, but Con A-treated cells did not show these changes. Instead, a highly electron-dense layer was observed in the outermost papillae, and the excess fibrous materials remained at the inside of the layer. These results suggest that an unknown molecule(s) recognized by Con A is essential for the diffusion of fibrous materials at the conjugation papillae, which is an indispensable step for gamete release during conjugation of the *C. psl.* complex.

## Introduction

Land plants (embryophyta) are believed to have evolved from ancestral charophycean algae (Karol et al., 2001). The charophyceans comprise five lineages (orders) of freshwater green algae: Charales, Coleochaetales, Zygnematales, Klebsormidiales, and Chlorokybales. Based on phylogenetic analyses, Zygnematales – or a clade consisting of the Zygnematales and the Coleochaetales – has been proposed as a sister group of land plants (Turmel et al., 2006; Wodniok et al., 2011).

The desmid *Closterium*, which belongs to Zygnematales, is the most successfully studied unicellular charophycean alga in terms of the maintenance of strains and sexual reproduction (Sekimoto et al., 2012, 2014a,b). Heterothallic strains of the *Closterium peracerosum-strigosum-littorale* complex (*C. psl.* complex) have two sexes: mating type plus (mt^+^) and mating type minus (mt^-^). Sexual reproduction readily occurs when cells of these two sexes are cultured together in nitrogen-depleted medium under light conditions. Recently, a technique for achieving reverse genetics in this complex has been developed (Abe et al., 2011; Hirano et al., 2015) and its genome project is now underway (Sekimoto et al., personal communication).

Two sex pheromones are essential for regulating the processes of sexual reproduction: protoplast-release-inducing protein (PR-IP) and PR-IP Inducer (Sekimoto et al., 1990, 1993). PR-IP is secreted from mt^+^ cells and induces the secretion of mucilage, sexual cell division for differentiation into gametangial cells (Akatsuka et al., 2006), and the release of protoplasts (gametes) from the gametangial cells (Sekimoto et al., 1990), whereas PR-IP Inducer is secreted from mt^-^ cells and induces the secretion of mucilage (Akatsuka et al., 2003), sexual cell division (Tsuchikane et al., 2003, 2005), and the expression of genes encoding two subunits of PR-IP (Sekimoto et al., 1994). Both gametangial cells make a sexual pair, form conjugation papillae, release their gametes, and form a zygote by the fusion of the gametes (Sekimoto et al., 2012); however, the mechanisms behind the pairing for zygote formation remain unclear.

From the results of cDNA microarray analyses, we identified 88 pheromone-inducible, conjugation-related, and/or sex-specific genes (Sekimoto et al., 2006). Among them, the function of the *CpRLK1* gene encoding receptor-like protein kinase was characterized (Hirano et al., 2015). CpRLK1 localized at conjugation papilla and the knockdown mt^+^ transformants of *CpRLK1* showed reduced competence for sexual reproduction and formed an abnormally enlarged conjugation papilla after pairing with the mt^-^ cells. Because CpRLK1 shares phylogenetic features with the CrRLK1L-1 subfamily, which is thought to act as a cell wall integrity sensor in higher plants, it is proposed that CpRLK1 is an ancient cell wall sensor and regulates the osmotic pressure of the cell for appropriate gamete release, a process required for successful conjugation.

To elucidate further the mechanisms of conjugation, especially the release and fusion of gametes in the *C. psl.* complex, we have focused on the lectins, carbohydrate-binding proteins. Generally, carbohydrate plays important roles in biological organization on the cell surface and cell-cell recognition phenomena. Hori et al. (2012) examined whether some lectins affected the conjugation processes of the *C. psl.* complex. Among 20 lectins tested, we conclude that a concanavalin A (Con A), which bind to mannose and glucose residues in carbohydrates and in *N*-linked glycan moieties of glycoproteins, is a certain candidate to understand the phenomena during fusion of gametes in *C. psl.* complex. Hori et al. (2012) reported that fluorescein isothiocyanate (FITC)-labeled Con A weekly bound to the surface of vegetative and nitrogen-deprived cells, however, binding of FITC-labeled Con A at conjugation papillae is quite remarkable and the addition of Con A completely inhibited zygote formation, indicating binding molecule(s) of Con A localized on the conjugation papillae is specifically involved in zygote formation and plays important roles during the process of conjugation in the *C. psl.* complex. However, the precise details of Con A inhibition during conjugation remain unclear.

In this study, we thus investigated the physiological and ultrastructural changes caused by Con A during the conjugation in the *C. psl.* complex. The results indicate that the Con A binding molecule(s) on conjugation papillae acts to disperse uncharacterized fibrous materials, which is indispensable for the release of gametes in the *C. psl.* complex.

## Materials and Methods

### Strains, Culture Conditions, and Preparation of Sexually Differentiated Cells

The strains of the heterothallic *C. psl.* complex used in this work were NIES-67 (mt^+^) and NIES-68 (mt^-^), obtained from the National Institute for Environmental Studies (Ibaraki, Japan). Cells were grown in 300-mL Erlenmeyer flasks containing 150 mL of nitrogen-supplemented medium (C medium^1^) at 23°C under a 16:8-h light:dark regime. Light from fluorescent lamps (FL40SSD; Toshiba, Tokyo, Japan) was adjusted to 28 μmol m^-2^ s^-1^ at the surface of the culture medium. To obtain sexually differentiated cells, mt^+^ and mt^-^ vegetative cells in mid-logarithmic phase (10 days of culture) were separately harvested by centrifugation and washed three times with nitrogen-depleted mating-inducing (MI) medium (Ichimura, 1971). Then, 7.2 × 10^5^ cells were inoculated into 72 mL of MI medium in a 300-mL Erlenmeyer flask and incubated for 24 h in continuous light (28 μmol m^-2^ s^-1^) (Sekimoto and Fujii, 1992).

### Inhibition of the Conjugation Processes by the Addition of Con A during Mating Culture

Cells at conjugation stages were obtained by mixing the respective sexually differentiated cells (5.0 × 10^3^ cells each in 1 mL of MI medium) in a test tube (1.75 cm in diameter, 13 cm long) and then incubated for 48 h under continuous light (28 μmol m^-2^ s^-1^). FITC-labeled Con A (Vector Laboratories, Burlingame, CA, USA) was added to cells at a final concentration of 7 μg mL^-1^ at each of 0, 8, 16, and 24 h after mixing of the cells. In parallel, mixed cells in the absence of Con A were also prepared and fixed using 0.6% (w/v) glutaraldehyde (Wako, Japan, Osaka) at these different periods of Con A addition. The population of sexual reproductive cells (conjugation pairs, gamete-releasing cells, and zygotes) was counted 48 h after the mixing.

### Localization of FITC-Labeled Con A

To monitor the developmental changes on conjugation papillae, 2.0 × 10^4^ cells mL^-1^ of sexually differentiated mt^+^ and mt^-^ cells were stained separately by Calcofluor White ST (American Cyanamid Co., Wayne, NJ, USA) at a final concentration of 0.1% (w/v) for 1 h under dark conditions. They were then harvested, washed three times with MI medium, and mixed together at a cell density of 5.0 × 10^3^ cells each in 1 mL of MI medium. After 16 h of incubation under continuous light, cells were harvested, washed three times with MI medium, and incubated in MI medium containing FITC-labeled Con A at a final concentration of 10 μg mL^-1^ for 1 h under dark conditions. Then, cells were washed three times with MI medium and fluorescence of the cells was observed using a fluorescent microscope (BX60; Olympus, Tokyo, Japan).

The localization of FITC-labeled Con A on conjugation papillae was detected as follows: cells at pairing stages (16 h after the mixing) were harvested, washed three times with MI medium, and resuspended in the same medium at a final density of 1 × 10^4^ cells mL^-1^. Then, both FITC-labeled Con A (final concentration: 10 μg mL^-1^) and Calcofluor White ST (final concentration: 0.1%) were added to the cells simultaneously. After 1 h of incubation under dark conditions, the fluorescence of the cells was detected using a confocal laser scanning microscope (FV500; Olympus).

### Transmission Electron Microscopy

Fluorescein isothiocyanate-labeled Con A was added to the cells at the pairing stage (16 h after the mixing) at a final concentration of 10 μg mL^-1^, followed by incubation for 4 h under continuous light. The cells were then harvested by centrifugation, prefixed in 2% glutaraldehyde in 0.1 M phosphate buffer or 0.05 M Cacodylate buffer (pH 7.2), and kept at room temperate for 2 h. Next, they were gently centrifuged, washed three times in this buffer, and postfixed in 1% OsO_4_ with the same buffer at 4°C overnight. After washing with the buffer, 1 × 10^5^ cells were collected by filtration using SUPREC-01 (Takara, Shiga, Japan). They were embedded in 2% low-melting-point agarose, dehydrated through an ethanol series, and passed through QY-2 (methyl glycidyl ether; Nisshin EM, Tokyo, Japan) and embedded in Quetol-812 mixture (Nisshin EM), followed by polymerization at 60°C for 48 h. Ultrathin sections of 60–70 nm were cut with an ultramicrotome, Ultracut S (Reichert-Nissei, Tokyo, Japan), stained with 4% uranyl acetate and 0.4% lead citrate, and observed with a TEM, JEM-1200EXS (JEOL, Tokyo, Japan), at 80 kV.

## Results

### Con A Inhibits the Process of Gamete Release or Later Stages in the *C. psl.* Complex

When both mt^+^ and mt^-^ cells of the *C. psl.* complex are mixed in nitrogen-depleted medium, the following processes of sexual reproduction are observed: (i) mucilage secretion, (ii) sexual cell division to form gametangial cells, (iii) pair formation between opposite mating type gametangial cells, (iv) formation of conjugation papillae, (v) release of protoplasts (gametes) from the cells, and (vi) fusion of the gametes to form a zygote (Ichimura, 1971; Sekimoto et al., 2012). We previously reported that Con A completely inhibits the zygote formation (Hori et al., 2012). Even though the results indicated that the target molecule(s) of Con A plays important roles in zygote formation, the details of the stage influenced by Con A have not been clarified.

**FIGURE 1:**
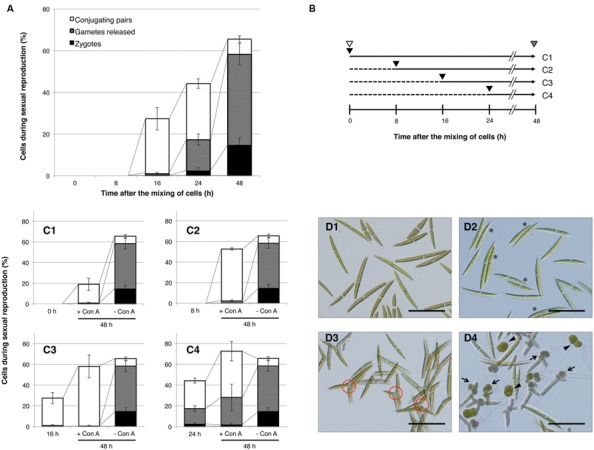
**Progress of sexual reproduction in the presence or absence of concanavalin A (Con A). (A)** Sexual reproductive stages in the absence of Con A. Independently prepared sexually differentiated mt^+^ and mt^-^ cells were mixed and incubated. At intervals shown on the *x*-axis, cells undergoing sexual reproduction (conjugating pair-forming cells, gamete-releasing cells, and zygotes) were counted (*n* = 3). **(B)** The experimental procedure to determine the developmental stages that are inhibited by Con A. Independently prepared sexually differentiated mt^+^ and mt^-^ cells were mixed (shown by white arrowheads) and incubated for 48 h. At 0, 8, 16, or 24 h after the mixing, Con A was added to the respective test tubes (black arrowhead). Populations of sexually reproductive cells were examined at 48 h after the mixing (gray arrowhead). Horizontal arrows with solid and dashed lines indicate periods of incubation with or without Con A, respectively. The numbers on the right correspond to the results shown in **(C1–4)**. **(C1–4)** Effect of Con A on the progress of sexual reproduction. Con A was added at 0 **(C1)**, 8 **(C2)**, 16 **(C3)**, or 24 h **(C4)** after the mixing of cells. The bar on the left in each graph shows the cell populations at a time of Con A addition. Bars at the center and right in each graph indicate the cell population after 48 h of incubation with or without Con A, respectively. White, gray, and black boxes show the cell populations in conjugating pairs, gamete-releasing cells, and zygotes, respectively. **(D1–4)** Photographs of mixed cells with or without Con A. **D1**: Initial cells (0 h after the mixing of cells). **D2**: Conjugating pairs (16 h after the mixing of cells). Conjugating pairs are shown by asterisks. **D3**: Cells after 48 h of incubation in the presence of Con A (Con A was added 8 h after mixing of the cells). Conjugation papillae are shown in red circles. **D4**: Cells after 48 h of incubation in the absence of Con A. Gamete-releasing cells and zygotes are shown by arrows and arrowheads, respectively. Scale bar: 100 μm.

First, we confirmed the temporal changes during the conjugation process in the *C. psl.* complex. Equal numbers of mt^+^ and mt^-^ cells, which had been separately induced in advance to undergo sexual differentiation in nitrogen-depleted medium, were mixed and then monitored for changes in the ratio of sexually reproductive cells at three developmental stages: conjugating pairs, gametes, and zygotes. As shown in **Figure 1A**, the mixed cells did not exhibit signs of sexual reproduction within the first 8 h; however, some of them had begun to form conjugating pairs by 16 h, followed by an increase in the release of gametes and zygote formation from 24 to 48 h.

Concanavalin A was added to samples at 0, 8, 16, and 24 h after the initial mixing and then comparisons to the proportion of sexually reproducing cells at 48 h were performed. The experimental procedure is outlined in **Figure 1B**, and the results are shown in **Figures 1C,D**. In the absence of Con A, about 66% of cells exhibited signs of sexual reproduction at 48 h after the mixing: 7, 44, and 14% of the cells formed conjugating pairs, released gametes, and formed zygotes, respectively (**Figures 1C,D4**).

When mixed cells, to which Con A had been added at 0, 8, and 16 h after the mixing, were incubated for 48 h (**Figure 1B**), they showed signs of sexual reproduction at rates of 19, 53, and 58%, respectively (**Figure 1C1–C3**), although the cells were mostly in the conjugating pair stage. Moreover, the conjugating pairs in the presence of Con A abundantly extruded conjugation papillae, but the release of gametes was markedly absent (**Figure 1D3**). These results clearly indicate that Con A affects the release of gametes, which is a prerequisite for cell fusion. In addition, no released gametes were observed in Con A-treated mixed cells by 16 h after extending incubation for 10 days (data not shown). This indicates that the addition of Con A does not delay the progression of gamete release, but instead completely inhibits it.

By comparison with the cells prior to Con A addition (0, 8, and 16 h after mixing), the proportion of conjugating pairs after 48 h of incubation increased from 0 to 18%, 0 to 51%, and 26 to 58%, respectively (**Figure 1C1–C3**). This indicates that Con A does not fully inhibit early processes of conjugation leading up to the formation of conjugating pairs. However, cells treated with Con A at 0 h exhibited reduced sexual reproduction compared with those at 8 and 16 h (**Figure 1C1**). Although this suggests that Con A exerts some effects on mixed cells during the early stages, this will be further elucidated in Section “Discussion.” Reduced zygote formation was also observed (**Figure 1C4**) when Con A was added at 24 h after the mixing, suggesting that it might affect cellular processes after gamete release.

### Behavior and Subcellular Localization of Con A-Target Molecules

**FIGURE 2:**
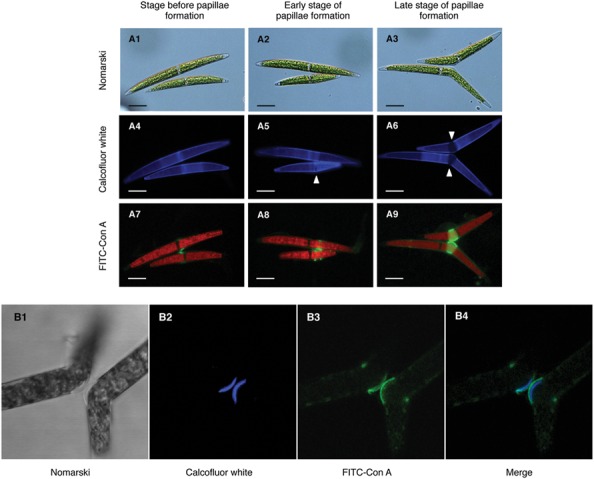
**Subcellular localization of Con A-binding molecule(s). (A1–9)** Development of conjugation papillae and subcellular localization of Con A binding molecule(s) during development. Both mating type cells were separately pulse-labeled by Calcofluor White prior to the mixing of cells. Fluorescein isothiocyanate (FITC)-labeled Con A was added to the mixed cells just before pair formation (8 h after the mixing of cells). **(A1–3)** Nomarski images of three developmental stages of papilla formation. **(A4–6)** Calcofluor White staining images. Novel unstained papillae (arrowhead) expanded during development. **(A7–9)** Localization of FITC-Con A binding molecule(s). Red signals in each cell represent auto-fluorescence of chlorophyll. Scale bar: 20 μm. **(B1–4)** Localization of Con A binding molecule(s) on conjugation papillae. Calcofluor White and FITC-labeled Con A were simultaneously added to mixed cells extruding the conjugation papillae. **(B1)** Nomarski image of conjugation papillae. **(B2)** Fluorescence by Calcofluor White. **(B3)** Fluorescence by FITC-labeled Con A. **(B4)** Merged image of B2 and B3.

In a previous study, we demonstrated that target molecule(s) of Con A mainly localized on the conjugation papillae during conjugation processes in the *C. psl.* complex (Hori et al., 2012). To monitor the developmental changes on conjugation papillae, Calcofluor White, which stains β-D-glucopyranose polysaccharides in cell walls, was used as a vital staining reagent. Both mating type cells were separately pulse-labeled by Calcofluor White and then mixed. Fluorescence on the whole cell surface and the central area was clearly detected before formation of the pair (**Figure 2A4**). After physical contact of the cells, narrow slit-like bands, which did not emit fluorescence signals, appeared near the base of the semi-cells (**Figure 2A5**, arrowhead). Upon enlargement of these bands, conjugation papillae appeared (**Figure 2A6**, arrowhead), namely, the conjugation papillae were synthesized *de novo* after pair formation. When FITC-labeled Con A was added to the mixed cells just before pair formation, the signals were first detected at the point of adhesion of the cells (**Figure 2A7**), then on the edge of developing papillae (**Figure 2A8**), and finally on conjugation papillae with a ring-like shape (**Figure 2A9**). These results indicated that the Con A binding molecule(s) was synthesized prior to papilla formation and accumulated around developing papillae.

Next, we investigated the exact localization of Con A binding molecules on conjugation papillae by confocal laser scanning microscopy. Calcofluor White and FITC-labeled Con A were simultaneously added at the time of conjugation papilla formation. As shown in **Figure 2B2**, Calcofluor White fluoresced intensively on the outer layers of conjugation papillae, indicating that the conjugation papillae consist of cell wall materials such as cellulose. Interestingly, fluorescence detected in the papillae did not exhibit contact between adhesion points in each mating type cell (**Figure 2B2**). Furthermore, signals derived from FITC-labeled Con A were detected on the outermost layer of the papilla (**Figure 2B3**), where the signals were not co-localized but contacted at adhesion points between the two mating type cells (**Figure 2B4**).

### Con A Inhibits the Release of Fibrous-Like Materials from Conjugation Papillae

As shown in **Figure 1C**, Con A inhibited the release of gametes from paired cells. To investigate the intracellular changes caused by Con A, we compared the ultrastructures in conjugation papillae with and without Con A.

**FIGURE 3:**
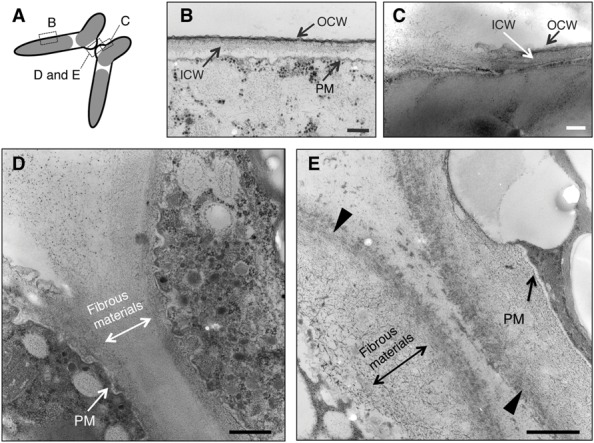
**Ultrastructural observations of paired cells using a transmission electron microscope (TEM). (A)** Illustration of TEM sections observed in this study. **(B)** *Closterium* cell wall consists of two layers: outer cell wall (OCW) and inner cell wall (ICW). Around the conjugation papillae, the OCW was disrupted **(C)** and fibrous-like materials were released from the papillae **(D)**. When Con A was added prior to cell fusion (16 h after the mixing), the fibrous-like materials accumulated in the conjugation papillae of respective paired cells. Increased electron density at the outermost layer was also observed (arrowhead) **(E)**. PM, plasma membrane. Bars: 250 nm **(B)**, 200 nm **(C)**, and 500 nm **(D,E).**

Sections obtained in the experiments are shown in **Figure 3A**. In the absence of Con A, two layers of cell wall showing different electron densities, high electron density (outer cell wall, OCW) and low electron density (inner cell wall, ICW), were observed in horizontal sections at the intermediate region of semi-cells (**Figure 3B**). At the basal regions of the conjugation papillae, disruption of the OCW and the release of fibrous-like materials from the ICW (or further inside the ICW) were detected (**Figure 3C**). Numerous vesicles of various sizes were detected in the cytoplasmic portion of the papillae, suggesting the active supply of unknown materials into the intercellular space (**Figure 3D**). The fibrous-like materials spread densely and subsequently filled the area between the papillae of the pairing cells. On the other hand, the fibrous-like materials did not accumulate in the intercellular space in the presence of Con A. Distinct highly electron-dense layer on the outermost papillae and accumulation of fibrous-like materials within the conjugation papillae were observed (**Figure 3E**).

From the aforementioned ultrastructural comparisons, it was indicated that the disruption of OCWs and the subsequent release of fibrous-like materials in conjugation papillae were indispensable for the release of gametes and that Con A binding molecules were possibly involved in the dispersion of such fibrous materials in the *C. psl.* complex.

## Discussion

We previously showed that Con A completely inhibited the zygote formation of the *C. psl.* complex (Hori et al., 2012). In the present study, we investigated the physiological and ultrastructural changes caused by Con A during conjugation processes in the *C. psl.* complex.

Normally, cells of both mating types form conjugation pairs by 16 h after mixing and then gamete release and zygote formation can subsequently be observed (**Figure 1A**). As shown in **Figure 1C1–C3**, mixed cells, to which Con A had been added by 16 h after the mixing, formed a sexual pair and conjugation papillae; however, subsequent processes were almost completely blocked. These results indicate that Con A does not affect the formation of sexual pairs and conjugation papillae but completely inhibits the later process of gamete release. However, we detected partial inhibition of the formation of conjugation pairs when Con A was added at the time of cell mixing (0 h). Hori et al. (2012) showed that Con A bound abundantly on conjugation papillae, but also slightly on the surface of vegetative and nitrogen-deprived cells. Indeed, both vegetative and sexual cell divisions were partially inhibited in the presence of Con A (data not shown). Therefore, we consider that Con A also binds to a molecule(s) that is essential for sexual cell division, and inhibits the pairing partly by inhibiting the formation of sexually competent gametangial cells.

On the other hand, the addition of Con A completely inhibited the processes after the formation of conjugation papillae (**Figure 1C1–C3**) and the FITC-Con A bound to the conjugation papillae remarkably (**Figure 2**), suggesting that Con A-binding molecule(s) on the conjugation papillae is critically responsible for the gamete release during the conjugation processes. In the absence of Con A, the disruption of OCWs and the diffusion of fibrous materials from the conjugation papillae were clearly observed (**Figures 3C,D**). In contrast, Con A-treated cells exhibited the highly electron-dense layer at the outermost papillae, and the excess fibrous materials remained at the inside of the layer (**Figure 3E**). These results clearly indicate that Con A blocks diffusion of the fibrous materials from the papillae.

**FIGURE 4:**
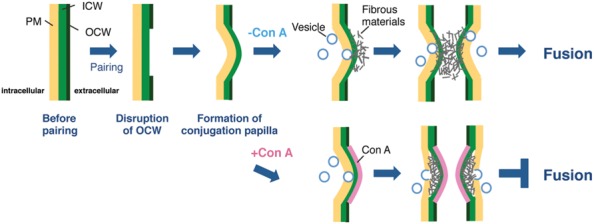
**Possible model for cell fusion in the *C. psl.* Complex.** Once mt^+^ and mt^-^ cells have formed a conjugation pair, the OCWs (OCW) at the point of adhesion of the two cells are disrupted. The cytoplasm of both paired cells is extruded from the thin wall area to form papillae by osmotic pressure. In parallel, fibrous materials, which are indispensable for gamete release, diffuse from the ICW to the tip of papillae with the supply of materials by vesicle transport. The target molecule of Con A is synthesized at adhesion sites prior to the formation of papillae and finally acts to unfold or refold the fibrous materials on the surface of papillae. In the presence of Con A, the highly electron-dense layer at the outermost papillae is appeared and the unfolding or refolding by the target molecule is inhibited; therefore, paired cells cannot complete gamete release and cell fusion.

From the fluorescence in the papillae stained by Calcofluor White (**Figure 2B2**), which recognizes β-D-glucopyranose polysaccharides, it was apparent that the conjugation papillae contained cell wall materials such as cellulose. Using a TEM, Pickett-Heaps and Fowke (1971) also reported that the conjugation papillae in *Closterium littorale*, a species related to the *C. psl.* complex, contained cell wall materials (Pickett-Heaps and Fowke, 1971). We also observed numerous vesicles in the cytoplasm inside the papillae (**Figure 3D**), probably due to active transport of the materials for papilla formation. These results suggest that the papillae are formed by rapid and considerable deposition of cell wall materials, accompanied by the rapid synthesis of cellulose microfibrils on the surface of the plasma membrane. Because of the co-localization of the fluorescence of Calcofluor White (**Figure 2B2**) and fibrous materials in the papillae (**Figure 3E**), the fibrous materials may be cell wall components. In addition, we also observed that the mutual contact area of two papillae is filled with fibrous materials in the absence of Con A (**Figure 3D**), suggesting the crossing of fibrous materials between the cells may be essential for cell fusion. In the case of Con A-treated cells, however, the diffusion of fibrous materials could not be observed (**Figure 3E**), suggesting that the diffusion is indispensable for the release of gametes and cell fusion. From these discussions, we consider that an unknown molecule(s) recognized by Con A may be involved in the unfolding or refolding of fibrous materials on the surface of conjugation papillae to achieve gamete release and cell fusion (**Figure 4**). At present, however, we have no information whether the binding of Con A might be a general obstruction to fibrous material release and such obstruction might be seen in the other life stage, such as vegetative cell division, or the obstruction is specific event and is only triggered on the conjugation papillae. Alternatively, the molecule may be an unknown signaling molecule for gamete release. In the *C. psl.* complex, a sex pheromone, PR-IP (Sekimoto et al., 1990), is known to be involved in gamete release from mt^-^ cells; however, no binding of Con A to PR-IP was confirmed by Western blotting (data not shown).

To identify the Con A-target molecules, two-dimensional polyacrylamide gel electrophoresis and western blotting with biotin-labeled Con A have been performed. In preliminary experiments, several spots have been abundantly detected in pairing-induced samples, comparing to the non-pairing samples (Supplementary Figure S1). One of them, having molecular mass of 75 kDa (red arrow), showed most apparent difference between two samples. These ConA-binding polypeptide species, that appear specific to induction of sexual pair formation, represent candidates for the mediation of the release of fibrous material. It will be necessary to identify these molecules in order to test this proposed function, for example by using our established knockdown procedure (Hirano et al., 2015).

## Author Contributions

JA, SH, and HS conceived of and designed the research. SH performed all experimental work. MS provided essential support for the TEM experiments. JA and HS wrote the manuscript.

## Conflict of Interest Statement

The authors declare that the research was conducted in the absence of any commercial or financial relationships that could be construed as a potential conflict of interest.

## Footnotes

1 http://mcc.nies.go.jp/02medium-e.html#c
