# Supplementary material for: Concanavalin A Disrupts the Release of Fibrous Material Necessary for Zygote Formation of a Unicellular Charophycean Alga, Closterium peracerosum-strigosum-littorale Complex
Source: Front Plant Sci. 2016 Jul 13;7:1040. doi: 10.3389/fpls.2016.01040 (PMC4942458; doi:10.3389/fpls.2016.01040)
Supplement: Supplementary file 1 [file Image_1.PDF]

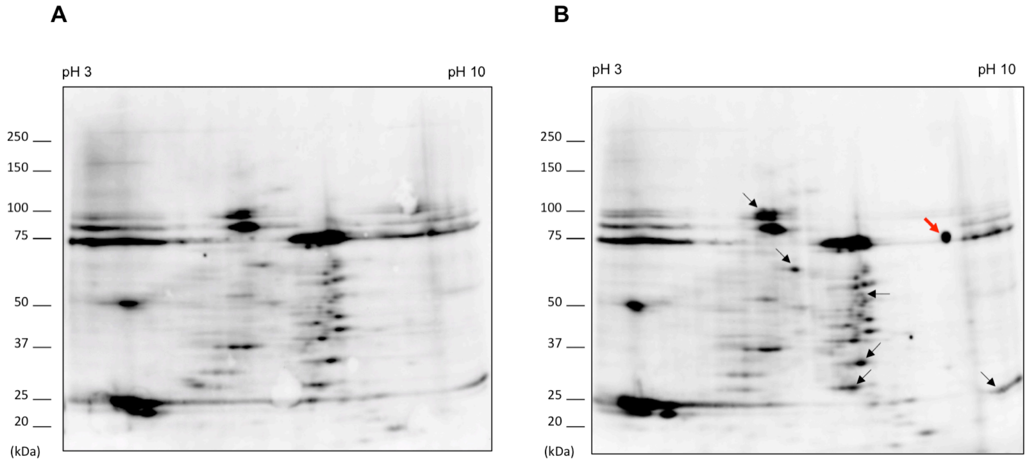

Supplementary Figure 1

Comparative detection of the Con A-recognized proteins of non-pairing cells (A) and pairing-induced cells (B). These proteins were separated by two-dimensional polyacrylamide gel electrophoresis and detected by biotin-labeled Con A. Arrows indicate the spots that are accumulated in pairing-induced cells comparing to the non-pairing cells. The spot indicated by red arrow is the most promising candidate among them.

Preparation of proteins and electrophoresis were performed as follows: Cells containing conjugating pairs (16 h after mixing of sexually differentiated mt^+ and mt^- cells in MI medium) were collected, washed three times with MI medium, and then resuspended in lysis buffer (7 M urea, 2 M thiourea and 4% CHAPS). As a control, non-mixing cells (16 h incubation of sexually differentiated mt^- cells in MI medium) was also prepared correspondingly. The cells were subjected to ultrasonic treatment using an ultrasonic disrupture (BIORUPTOR, Cosmo Bio, Tokyo, Japan). The homogenates were centrifuged at 1,000 x *g* for 10 min, and then protein concentration of the supernatants were quantified with Bradford method. The prepared proteins were loaded into the IPG strips (7 cm, pH from 3.0 to 10.0, Bio-rad, Hercules, CA, USA) for the first dimensional isoelectric focusing (IEF). After the IEF, the IPG strips were then subjected to SDS–PAGE. After electrophoresis, the proteins in the gel were transferred electrophoretically onto a nitrocellulose membrane (Optitran BA-S 85, Whatman, Dassel, Germany) and probed with biotin-labeled Con A and horseradish peroxidase conjugated streptavidin (Vector Laboratories, Burlingame, CA, USA). The Con A-binding proteins were detected by chemiluminescence of substrate (ImmunoStar LD, Wako, Osaka, Japan) using a Versadoc (Bio-rad).
